# Novel insights into host-fungal pathogen interactions derived from live-cell imaging

**DOI:** 10.1007/s00281-014-0463-3

**Published:** 2014-11-15

**Authors:** Judith Bain, Neil A. R. Gow, Lars-Peter Erwig

**Affiliations:** Aberdeen Fungal Group, Institute of Medical Sciences, University of Aberdeen, Aberdeen, AB25 2ZD UK

**Keywords:** Macrophage, Neutrophil, Candida, Fungi, Phagocytosis, Live imaging

## Abstract

The theoretical physicist and Nobel laureate Richard Feynman outlined in his 1959 lecture, “There’s plenty of room at the bottom”, the enormous possibility of producing and visualising things at smaller scales. The advent of advanced scanning and transmission electron microscopy and high-resolution microscopy has begun to open the door to visualise host-pathogen interactions at smaller scales, and spinning disc confocal and two-photon microscopy has improved our ability to study these events in real time in three dimensions. The aim of this review is to illustrate some of the advances in understanding host-fungal interactions that have been made in recent years in particular those relating to the interactions of live fungal pathogens with phagocytes. Dynamic imaging of host-pathogen interactions has recently revealed novel detail and unsuspected mechanistic insights, facilitating the dissection of the phagocytic process into its component parts. Here, we will highlight advances in our knowledge of host-fungal pathogen interactions, including the specific effects of fungal cell viability, cell wall composition and morphogenesis on the phagocytic process and try to define the relative contributions of neutrophils and macrophages to the clearance of fungal pathogens in vitro and the infected host.

## Introduction

Fungal infections contribute substantially to human morbidity and mortality. Over 1.5 billion people suffer from fungal infections. Many of these are chronic infections of the skin and nails, but of great concern are the millions of people per year that acquire life-threatening invasive infections which can have mortality rates over 50 %. Mortality rates for serious fungal infections outstrip nearly all those caused by pathogens such as Methicillin resistant *Staphylococcus aureus* (MRSA) and *Escherichia coli* but unlike these, none of the top four fungal pathogen genera (*Candida*, *Aspergillus*, *Cryptococcus* and *Pneumocystis*) are household names. In fact, more people die from fungal infections than malaria or tuberculosis. A majority of fungal-related deaths occur in developing countries, particularly in sub-Saharan Africa, where for example, *Cryptococcus* kills more than 600,000 HIV infected patients per annum [1, 2]. The predisposing factors leading to disseminated fungal infection include chemotherapy, the use of immunosuppressive drugs and long-term use of broad-spectrum antibiotics that affect the equilibrium of the microbial flora. Prolonged hospitalisation and use of medical indwelling devices and central venous catheters further increase the risk of disseminated fungemia [3]. Clinical cases of disseminated candidiasis in patients with neutropenia or other conditions, where the function and numbers of leucocytes are compromised highlight the importance of innate immunity in the control of systemic fungal infections.

Various fungal pathogens have evolved the ability to successfully colonise diverse niches of the human body, effectively preventing recognition by the host immune system (for example by binding negative regulators of the complement cascade to inhibit complement activation), and therefore constitute part of the normal microbial flora of the gut, oral and vaginal cavity in healthy individuals [4]. Another common evasion mechanism of fungal pathogens involves the concealment of cell wall pathogen-associated molecular patterns (PAMPs). One of the best examples is the extracellular polysaccharide capsule of *Cryptococcus neoformans*, which covers the cell wall, preventing recognition and uptake by host cells [5]. Other pathogens mask underlying PAMPs, including β-glucan by molecules that are not recognised by PRRs, such as the outer cell wall α-glucan layer of *Histoplasma capsulatum* or the external hydrophobin layer of *Aspergillus fumigatus*-resting conidia [6, 7]. Concealment can also be achieved by morphogenesis such as the hyphae of *Candida albicans* that do not display β-glucan on the surface [8].

## The innate immune response to fungal infection in vitro

An essential component of the innate response to fungal infection involves macrophages and PMNs recognising pathogen-associated molecular patterns (PAMPs) present in the fungal cell wall through pattern recognition receptors (PRRs) localised on the phagocytic cell membrane, endosomes and cytoplasm [9, 10]. Engagement of these receptors enables the phagocyte to directly engulf and destroy fungal cells within the phagolysosome using a number of oxidative and non-oxidative mechanisms including the production of toxic reactive oxygen and nitrogen species (ROS and RNS), expression of various antimicrobial peptides and the activity of hydrolytic enzymes [11]. In response, fungi that have evolved to resist phagocyte-associated stresses, including reactive oxygen and nitrogen species (ROS/RNS) [12, 13]. For example, *C. albicans* encodes a catalase and six superoxide dismutases; unusually, three Sod enzymes (Sod4–6) are secreted and detoxify extracellular ROS produced by macrophages [14, 15]. It is possible that some of these processes evolved outside the context of pathogen-human interactions in ancient associations that evolved in the context of predation by unicellular soil phagocytes such as amoebae, that also deploy oxidative mechanisms to destroy their microbial prey [16].

PAMP-PRR interactions also facilitate indirect killing of *Candida* by triggering the induction of proinflammatory cytokines and chemotactic factors which serve to activate other arms of the host immune system and aid in the clearance of *Candida* from the body [9, 11]. Extensive work has been carried out identifying the PRRs and downstream-signalling pathways that are involved in phagocyte recognition of fungal cells [9, 17]. These studies have identified the Toll-like receptors (TLRs), C-type lectin receptors (CLRs) and Nod-like receptors (NLRs), as the major PRRs involved in *C. albicans* PAMP recognition [18, 19]. Species specific differences between the expression of neutrophil and macrophage C-type lectin receptors in mouse and man are likely contributors to the fungicidal potential of neutrophils and macrophages [19]. Our studies, and those of others, have revealed how the overall phagocytic process in macrophages and PMNs is affected by fungal cell wall composition, morphogenesis, and species [20–22]. For example, Keppler-Ross et al. demonstrated that J774.1 macrophages preferentially phagocytosed *C. glabrata* and *Saccharomyces cerevisiae* over *C. albicans*, and that these macrophages displayed a strong preference for *C. albicans* yeast cells rather than hyphal cells [20].

**Fig. 1:**
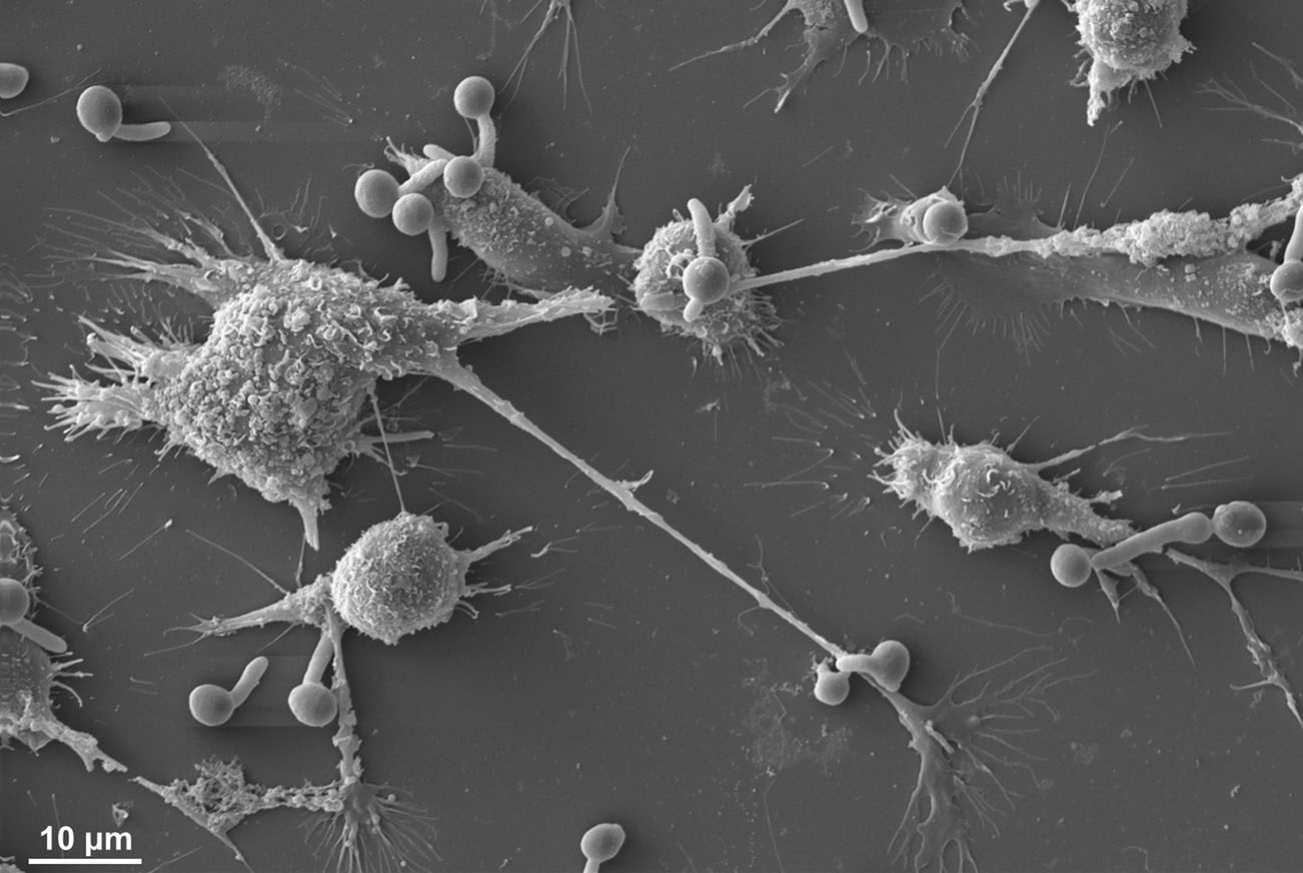
Scanning electron micrograph of RAW264.7 macrophages interacting with *C. albicans*. Fixed cells were visualised by SEM following 1 h of incubation, revealing long and ultrathin projections emanating from phagocytes. With thanks to Lucinda Wight, University of Aberdeen. Scale is shown at the *bottom left*

**Fig. 2:**
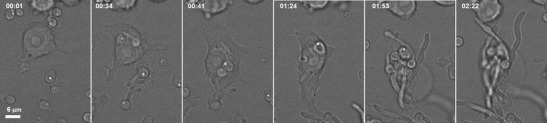
Sequence of selected frames from time lapse DIC imaging of J774.1 murine cell line macrophages phagocytosing live *C. albicans*. Images were captured at 1 min intervals for 3 h using an UltraVIEW VoX spinning disc microscope (Nikon, Surrey, UK) and Volocity software (Improvision, PerkinElmer, Coventry, UK). The time of image acquisition is noted in the top left of each panel (hh:mm). Scale is shown in the *bottom left* of the first panel. Over the period shown, the macrophage internalises at least 5 *C. albicans*, which continue to extend their hyphae, rupturing the macrophage by 1 h 53 min (*fifth panel*) allowing escape of fungi (*last panel*)

**Fig. 3:**
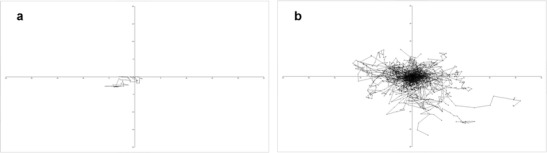
Phagocyte migration tracking charts. J774.1 macrophages pursuing live *C. albicans* were imaged with time lapse using a Nikon UltraVIEW VoX spinning disc microscope and the movies were analysed using Volocity software to determine macrophage migration, directionality and velocity from 1 min interval data, taken from the first 1 h period of incubation. The migratory path of a single macrophage is depicted (**a**) and the paths of 80 macrophages are shown, with starting positions all set to the zero position relative to *x* and *y* (**b**)

Our own work highlights the importance of fungal cell wall composition for phagocytosis. *C. albicans* deficient in cell wall *N*- and *O*-glycans negatively affected the capacity of PMNs to phagocytose and kill the fungus [22]. In contrast, macrophages preferentially ingested *O*- and *N*-linked mannan-deficient mutants but showed a reduced ability to ingest phosphomannan-deficient strains [22]. Observations of fixed cells give a snapshot of interactions at that point in time (Fig. 1); and importantly, in this study by McKenzie et al., [22], it was becoming apparent that the macrophages’ ability to ingest fungal cells was not directly linked to the phagocytes’ ability to kill the ingested pathogen. These studies are informative but highlight the need for novel technologies that go beyond assessing phagocytosis as a single event. Dynamic live-cell imaging enables the description of host-pathogen interactions as a series of individual stages: migration, recognition, engulfment, phagosome maturation and ultimately killing of the host cell or the ingested pathogen which are affected differentially when interacting with different cell types or mutants. Host cells and pathogens can be studied from continuous image sequences over extended time periods, providing spatial and temporal information on a broad range of dynamic processes (Fig. 2). Persistence and replication of fungal pathogens that are resistance to phagosomal degradation have been visualised by time lapse imaging of live cells, for example following phagocytosis of *C. glabrata*, which persist for 2–3 days before lysis of the monocyte-derived macrophages in which they reside [23]. A study by Lewis et al. utilised widefield live video microscopy [24] to dissect minute-by-minute the phagocytosis process of *C. albicans* by macrophages and developed a method to determine phagocyte migratory kinetics (Fig. 3), with which they showed migration towards *C. albicans* is dependent on the glycosylation status of the fungal cell wall, but not cell viability or morphogenic switching from yeast to hyphal forms [25]. This study also demonstrated that the rate of engulfment of *C. albicans* tethered to the macrophage surface is significantly delayed for glycosylation and yeast-locked morphogenetic mutant strains, but enhanced for non-viable cells [25]. Engulfment of bound *C. albicans* by macrophages is also influenced by morphology, as hyphal cells were engulfed at a slower rate than yeast cells, especially those with hyphae in excess of 20 μm, but there was no correlation between hyphal length and the rate of engulfment below this threshold. Furthermore, this study revealed that spatial orientation of the hyphae and whether hyphal *C. albicans* attached to the macrophage via the yeast or hyphal end were also important determinants of the rate of engulfment [25]. Such analyses of the influence of geometric apposition of irregular-shaped pathogens encountered by host cells could not be conducted without live imaging. Recent work using similar methodology studying the interaction of the important plant and opportunistic human pathogen *Fusarium oxysporum* with macrophages revealed remarkable similarities with the findings described for *C. albicans* above [26]. The study shows that murine macrophages efficiently migrate towards and internalise *F. oxysporum* germlings. The average engulfment time of bound *F. oxysporum* was almost identical to that reported in *C. albicans*. Furthermore, the number of engulfed fungal germlings crucially affected the survival of the macrophage. The vast majority of macrophages that internalised 4 or more germlings were killed by *F. oxysporum*, whereas less than 50 % of those were killed engulfed less than 4 germlings. Both *C. albicans* and *F. oxysporum* appeared to induce the majority of macrophage cell death by hyphal-mediated piercing of the macrophage cell membrane, although any contribution of the recently described mechanism of pyroptosis remains to be determined [27, 28]. Furthermore, intraphagosomal *F. oxysporum* germling formation inhibited macrophage mitosis at the level of cell separation in keeping with previous findings made for the fungal pathogens *C. neoformans*, *Candida krusei* and *C. albicans* [29–31]. Successful mitosis of macrophages carrying fungal cells may represent a route to potentially generate a non-infected innate immune daughter cell; therefore fungal interference with host mitosis counters this mechanism. A further phenomenon that could only be revealed by live visualisation of host-pathogen interactions is termed “vomocytosis” or non-lytic expulsion of fungal cells, which was first described by the groups of Casadevall and May for yeast cells of *C. neoformans* following phagocytosis by macrophages [32, 33]. This is now being increasingly recognised as a ubiquitous phenomenon occurring for many fungal pathogens including *C. albicans* [34]. The clinical relevance of this phenomenon is difficult to determine and warrants further investigation as non-lytic expulsion of fungal cells may benefit the macrophage in terms of avoiding lysis, but conversely may also benefit the fungus as it escapes the microbicidal environment of the phagosome. Following the discovery of non-lytic exocytosis of *C. neoformans* from phagocytes, lateral transfer between host cells was reported, adding to the repertoire of infection mechanisms utilised by this fungus [35]. *Cryptococcus* entry across the blood-brain barrier can occur by direct invasion of endothelia; however, there remains the possibility that transit to the central nervous system be facilitated within a macrophage “Trojan horse” [36].

Actin and phosphoinositide dynamics of mature phagosomes containing *C. albicans* were investigated using live imaging and revealed dynamic associations around hyphal phagosomes [37]. These observations may be interpreted such that actin-recruitment is a mechanism favoured by the pathogen to assist fungal escape, as is employed by other pathogens, including the comet tails elicited by *Listeria monocytogenes* from host actin [38]. On the contrary, actin polymerisation may be a host-driven mechanism to facilitate maturation of the phagosome [39]. Live imaging of clathrin-fluorescent protein fusions around phagosomes following the uptake of live *C. albicans* into epithelial cells has revealed that this occurs through hijacking of host clathrin-mediated endocytosis; underlining the manipulation of host cells at the gut mucosal habitat where commensal *C. albicans* can be found [40]. Live imaging of actin cages around phagosomes containing *Cryptococcus* prevents the exit of these fungi via the vomocytosis mechanism described above [41] and further exploration using the four-dimensional imaging (3D over time) will be a powerful tool to investigate these dynamics.

## Contributions of neutrophils and macrophages to the clearance of fungal pathogens

An important first line of defence against *Candida* infections is provided through the recruitment of professional phagocytes, such as polymorphonuclear cells (PMNs) and macrophages, to the site of infection [18, 25, 42]. Studies to date suggest a dominant role for the PMNs [43, 44] and this is reflected in the observation that neutropenic patients and patients with peroxidase-deficiency are highly susceptible to invasive candidiasis [45, 46]. In particular, patients with chronic granulomatous disease, displaying impaired neutrophil function, are especially prone to systemic fungal infections [47]. The role of macrophages in combating fungal infection is exemplified in studies in mice that showed that clodronate-induced depletion of mononuclear phagocytes results in accelerated tissue fungal proliferation and increased mortality [48], and that neutrophil depletion does not adversely affect the innate immune control of *Candida* in blood [49]. As mentioned above, neutrophils are thought to be the dominant phagocyte combating fungal infections as it was thought that *C. albicans* is unable to form filaments within neutrophils, and studies from the Fink lab show that *C. albicans* and *S. cerevisiae* are deprived of amino acids within neutrophil phagosomes [50]. Interestingly, recent work by Ermert et al. revealed that murine neutrophils exhibited a significantly lower ability to kill *C. albicans* than their human counterparts. Strikingly, *C. albicans* yeast cells formed germ tubes upon internalisation by murine neutrophils, eventually rupturing the neutrophil membrane and thereby, killing the phagocyte [51]. In contrast, the same study showed that growth and subsequent escape of *C. albicans* are blocked inside human neutrophils, and the authors suggest that this might be a result of higher levels of the respiratory burst enzyme myeloperoxidase (MPO) activity and the presence of α-defensins. The importance of cell wall mannans for the recognition and uptake of *C. albicans* by human PMNs was exemplified in a study by Sheth et al. that showed *N*- and *O*-glycosylation-deficient mutants were attenuated in binding and phagocytosis, and this was associated with reduced killing of *C. albicans* yeast cells. In the same study, no differences were found in the production of MPO and the neutrophil chemokine IL-8 in PMNs exposed to control and glycosylation-deficient *C. albicans* strains [52]. Furthermore, fungi and bacteria induce the formation of neutrophil extracellular traps (NETs) by activated PMNs, but not macrophages, which can entrap and kill fungi and bacteria extracellularly [53]. The antimicrobial heterodimer calprotectin is released in NETs as the major antifungal component. Absence of calprotectin in NETs resulted in complete loss of antifungal activity in vitro and in *C. albicans* in vivo infection models indicated that NET formation is a hitherto unrecognised route of calprotectin release and crucial for the clearance of infection [53].

**Fig. 4:**
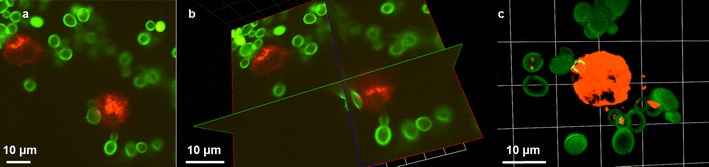
Three dimensional (3D) imaging of RAW264.7 macrophages interacting with *C. albicans*. FITC-stained *C. albicans* were coincubated with Lysotracker Red-stained macrophages and 64 slice z-stacks were captured at 1 min intervals for 1 h using a Nikon UltraVIEW VoX spinning disc microscope with Volocity software for image analysis. Images represent a single time point with interactions between macrophages and *C. albicans*, shown in extended focus (**a**), XYZ (**b**) and with 3D opacity rendering (**c**). Scale is shown at the *bottom of each panel*

Dynamic live-cell imaging can contribute to our understanding of the relative contributions of neutrophils and macrophages in the phagocytosis of fungal pathogens in vitro and may provide some clues with regard to the role phagocyte subsets in murine models and human disease. Spinning disc confocal microscopy is rapidly emerging as the technique of choice for the investigation of living cell-cell interactions. Modern commercial instruments and high-performance camera systems are capable of providing high-acquisition speeds with acceptable contrast and minimal photobleaching at the low-light levels available with this technique, facilitating rapid acquisition of images in four dimensions (Fig. 4).

A recent study employing spinning disc confocal microscopy assessed stage-specific *C. albicans* phagocytosis by human monocyte-derived macrophages and human PMNs in isolation with *C. albicans* and when both phagocyte subsets were present. Surprisingly, this study showed that when only one immune cell type was present, macrophages, not PMNs, had the greater capacity for *C. albicans* uptake despite engulfing fungal cells at a lower rate after establishing cell-cell contact [54]. However, when both cell types were present, macrophages and PMNs contributed approximately equally to the clearance of *C. albicans*. The study illustrates that uptake rates are affected not only by the fungal/phagocyte ratio but also by the proportion of the phagocyte subsets encountered. In this context it is important to point out that the nature of mononuclear phagocytes studied has significant impact on in vitro findings; the human monocyte-derived macrophages, mouse bone marrow-derived macrophages and resident macrophages isolated from the kidney have potent antifungal activity ex vivo [54–56], whereas thioglycollate-elicited peritoneal macrophages do not [57].

Furthermore, the live-cell imaging study by Rudkin et al. [54] showed that in vitro PMNs not only migrated more rapidly than macrophages towards *C. albicans* cells but also engulfed *C. albicans* cells at a significantly higher rate once cell-cell contact was established, and therefore mediate the majority of early *C. albicans* yeast cell uptake when both phagocyte subsets are present. As a consequence, macrophages encounter a higher proportion of hyphal cells, which they engulf at a lower rate. Both uptake of a higher proportion of hyphal *C. albicans* cells and increased hyphal length at the time of uptake is reportedly associated with host cell death [22, 25, 58].

Interestingly, in vitro studies using live-cell imaging show that macrophages can sense fungal cells at distances in excess of 15 μm and suggest shedding of fungal cell wall components may elicit a chemotactic response [25]. Differential migration of PMNs towards phenotypic variants of *C. albicans* perhaps underlines the importance of recognising intrastrain pathogenic phenotypes (“white” cells) as distinct from phenotypes more representative of commensal cells (“opaque” cells) to avoid inappropriate proinflammatory responses [59].

## Imaging host-pathogen interactions in the infected host

In vivo, the contribution of phagocyte subsets to overall uptake would also be influenced further by factors such as chemokine and cytokine concentrations and spatial considerations such as location of the fungal infiltrate, the numbers of phagocytes and accessibility to the infection sites. Studies in experimental models where neutrophils have to migrate successfully to the site of infection to come in contact with and kill fungal cells suggest that neutrophils are protective only when they accumulate early within the first 24–48 h after infection, as subsequent neutrophil recruitment does not confer additional survival benefit [49]. The different rates of neutrophil recruitment in organ systems in the mice may explain the organ specific pathology and disease progression [60], e.g. the liver and spleen, are able to recruit significant numbers of neutrophils and are able to control fungal proliferation and prevent filamentation. In contrast, the lack of efficient signals for rapid neutrophil recruitment in the kidney is associated with the inability of the organ to control fungal overgrowth and fungal abscess formation [60]. Therefore, defining the signals that drive neutrophil and macrophage recruitment to infectious sites in vivo is an important area for future research. The differences between macrophages and neutrophils as well as the apparent species specific differences between murine and human neutrophils have important implications for the use of experimental models to study systemic fungal infections. Mice are not the natural host for *Candida* species [61], and a variety of invertebrate and vertebrate hosts are used today to study disseminated candidiasis [62]. One example is the zebrafish larva model which enables non-invasive live visualisation of host-pathogen interactions [63]. The features of this system which are particularly amenable to live-cell microscopy include the transparency of the larva, which can be used at early stages of life without licencing restrictions [63, 64]. Zebrafish larval models allow fundamental questions pertaining to host-pathogen in vivo interactions to be addressed. Elegant work using this model by Brothers et al. visualised the cellular impact of loss of host-phagocyte NADPH oxidase activity on both host and pathogen, finding that it is the primary cause of oxidative stress in fungi, and that it limits filamentous growth [65]. Recently, the zebrafish model has been extended to demonstrate the different contributions of macrophages and neutrophils during infection with *A. fumigatus* [64].

Nonetheless, mammalian model organisms are most closely related to human biology and remain essential to understand initiation and progression of disease and allow for the development and evaluation of novel diagnostics and therapies [66], Thus, infection of mice by intravenous injection is the most widely accepted experimental infection model to study mycoses, offering advantages, such as high reproducibility, convenient handling and the availability of mouse strains with deletion of essential components of the immune system [66].

An exciting advance in the understanding of host responses to fungal infection have come from imaging live or newly extracted host tissue. Such methods have the advantage of retaining near native physiological conditions, which can be difficult to emulate during in vitro experimentation. In addition, dimensionality of host niche infection sites has been eloquently shown to impact the activity of neutrophils and macrophages phagocytosing *C. albicans* or *A. fumigatus* [67]. Live imaging was conducted to follow the dynamic movement and activities of host cells in response to fungi in either 2D or 3D settings, recapitulating the native sites of alveolar space or micro abscesses, respectively [67]. This study highlights the benefits of examining host-fungal interactions in native settings; however in doing so, resolution may be compromised. Non-invasive imaging into ear tissue of anaesthetised mice previously infected intradermally with a strain of *C. albicans* constitutively expressing eGFP has revealed in vivo morphogenesis and the formation of microabscesses within the same animal imaged on consecutive days [68]. These imaging methods add valuable information alongside clinical scoring of disease seen in live animals although deeper tissue requires histological examination following the sacrifice of animals. Whole animal in vivo imaging of infection by *C. albicans* is feasible using strains engineered to express bioluminescence [69]. However, some limitations have arisen from the necessity to co-inject the substrate for luciferase; unlike bacteria, eukaryotic cells require exogenous substrate. For example, the fungal cell wall is recalcitrant to the efficient uptake of substrate, with concomitant reduction in light emission, hampering efforts to visualise fungi within deeper tissue sites of infection. Some evidence of autoluminescence of the co-injected substrate has been reported, although with development, bioluminescence holds promise for the in vivo imaging of various fungal infections [70]. Recently, bioluminescent *C. albicans* infection in mice revealed an unexpected *Candida* reservoir in the gall bladder following treatment with antifungal therapy, which otherwise cleared infection, therefore live imaging can be informative of anatomical infection progression over a time span [71]. Bioluminescence emission by fungi has been successfully used in the work of Vande Velde et al., who have investigated *C. albicans* biofilms in live animals by combining a subcutaneous catheter model in mice with imaging of bioluminescence [72]. Biofilm formation of indwelling and prosthetic devices represent a significant clinical problem, therefore the ability to image biofilm development over time within in vivo settings will be vital in advancing therapeutics that can counter biofilms. Detailed imaging of live *C. albicans* biofilms with peripheral blood mononuclear cells (PBMCs) has revealed dynamic interactions; a detailed understanding of which will be fundamental to tackling this clinical problem [73]. Although the work was carried out in vitro, the interactions are captured at high resolution both temporally and spatially, offering new insight on a relatively unexplored field. Multiphoton microscopy offers the highest resolution imaging within an in vivo setting, at depths of up to 1 mm with lower light toxicity than confocal microscopy. Use of two-photon microscopy to study *A. fumigatus* infection of mouse lungs in situ can be done by intratracheal administration of an *A. fumigatus* dose, with 7 h used as the period given in which to allow the infection to establish. Mice were then euthanised and the chest cavity opened to permit imaging of the lungs, revealing phagocytosis of conidia and NETS production by neutrophils in a close-to native physiological setting [74, 75]. One limitation of this method is that absence of blood circulation, post mortem, prevents further influx of neutrophils during the imaging time. Multiphoton microscopy has been performed on human skin from patients carrying dermal fungal infections, thereby potentially offering patients a non-invasive “optical biopsy” which could expedite the identification of fungal infection, bypassing the necessity for culture-based identification [76]. In vivo imaging, therefore balances the ability to image within true-state physiological settings, against the very fine detail of interactions that can be visualised by in vitro live-cell imaging methods.

## Novel imaging technologies applied to fungal infection

Observations of the migratory kinetics of host cells in response to fungal pathogens are amenable to extrapolated investigation, for example by applying computational modelling to image sequences, as was done by Tokarski et al. [77]. This study was aimed at revealing the hunting strategies of neutrophils targeting *A. fumigatus* conidia, which upon inhalation, must be intercepted and inactivated before germination of destructive filaments are produced within lung tissue.

New developments in the field of host-fungal interactions may be driven by application of “optical tweezers”, a method which creates an optical trap using a focussed laser beam for the capture and movement of tiny particles. Tam et al. [78] demonstrated how this method could be utilised in the dissection of interactions between phagocytes and target particles including *C. albicans* and *A. fumigatus* for exquisite control and spatial and temporal manipulation of cellular components yielding exact observations of phagocytosis. For example, a fungal cell can be physically juxtaposed with a phagocytic cell, with live imaging conducted in parallel, to capture phagocytic events that followed. By combining optical trapping of particles with spinning disc microscopy and environmental control of specimens in line with physiological temperature and CO_2_ levels, these studies are an exciting step into an area which hands researchers the ability to place particles at the right place, at the right time.

Ultrafine resolution of host-fungal interactions can be examined with the use of atomic force microscopy (AFM). This method utilises a cantilever probe for scanning of samples to reveal nanometre resolution and ultrafine manipulation or measurement of interactions. AFM has been applied to address host-fungal interactions, including cellular ultrastructure during phagocytosis of *C. albicans* by macrophages [79]. While live-cell experiments can be conducted by AFM, high-quality resolution of surface topography comes from gentle fixation of the cells [79].

## Imaging fungal interactions with other innate immune cells

This review aims to provide a conceptual outline of the advanced made by novel imaging technologies in understanding the temporal dynamics of host-pathogen interactions rather than providing a complete review of the existing literature, and we apologise to those whose important relevant contributions are not cited. It is beyond the scope of this review to describe in detail imaging driven advances in understanding fungal interactions with host cells other than neutrophils and macrophages. Nonetheless, live-cell imaging has revealed novel host mechanisms for dendritic cell interactions with fungal particles including a description of the “fungipod”, a pseudopodial protrusion emanating from immature DCs [80]. These structures are dependent on the presence of mannose receptor and discriminate *Candida* species, exhibiting a preference for *C. parapsilosis* over *C. albicans* and *C. tropicalis* as a prerequisite to induce formation. Furthermore, fungipodia form in the presence of larger particles, ~5 um, are actin-driven structures and can persist for several hours; with intriguing possibilities of what role such structures may undertake during interactions between the host cell and the bound fungal particle [80]. Unexpected interactions have also been captured for natural killer cells using live imaging of *C. albicans*. Phagocytosis leads to activation of natural killer cells representing a novel route for immune activation [81].

## Conclusions

“Seeing is believing”. Live imaging methods have brought to light many interesting and sometimes unexpected findings in how fungi interact with host cells. Visualising these events provides incontrovertible evidence of hitherto unknown phenomena, for example non-lytic expulsion, lateral transfer, inhibition of mitosis and formation of giant cells. Imaging phagocytosis of fungal cells by host phagocytes illustrates clearly that phagocytic efficiency is dependent on the nature and origin of the innate immune cell, the phagocyte fungal cell ratio and the proximity and migratory capacity of the host towards the fungal cell. Furthermore, it absolutely establishes that the temporal dynamics of these processes rather than findings at selected endpoints are crucial to the overall outcome of the host-pathogen interaction. Sophisticated imaging combined with advanced image analysis tools and genetic manipulation of the host and the pathogen will play an important role in addressing key challenges in fungal immunology.
